# An Oxidoreductase AioE is Responsible for Bacterial Arsenite Oxidation and Resistance

**DOI:** 10.1038/srep41536

**Published:** 2017-01-27

**Authors:** Qian Wang, Yushan Han, Kaixiang Shi, Xia Fan, Lu Wang, Mingshun Li, Gejiao Wang

**Affiliations:** 1 State Key Laboratory of Agricultural Microbiology, College of Life Science and Technology, Huazhong Agricultural University, Wuhan 430070, P. R. China

## Abstract

Previously, we found that arsenite (As^III^) oxidation could improve the generation of ATP/NADH to support the growth of *Agrobacterium tumefaciens* GW4. In this study, we found that *aioE* is induced by As^III^ and located in the arsenic island near the As^III^ oxidase genes *aioBA* and co-transcripted with the arsenic resistant genes *arsR1*-*arsC1-arsC2-acr3-1*. AioE belongs to TrkA family corresponding the electron transport function with the generation of NADH and H^+^. An *aioE* in-frame deletion strain showed a null As^III^ oxidation and a reduced As^III^ resistance, while a *cytC* mutant only reduced As^III^ oxidation efficiency. With As^III^, *aioE* was directly related to the increase of NADH, while *cytC* was essential for ATP generation. In addition, cyclic voltammetry analysis showed that the redox potential (ORP) of AioBA and AioE were +0.297 mV vs. NHE and +0.255 mV vs. NHE, respectively. The ORP gradient is AioBA > AioE > CytC (+0.217 ~ +0.251 mV vs. NHE), which infers that electron may transfer from AioBA to CytC via AioE. The results indicate that AioE may act as a novel As^III^ oxidation electron transporter associated with NADH generation. Since As^III^ oxidation contributes As^III^ detoxification, the essential of AioE for As^III^ resistance is also reasonable.

Arsenic (As) is a toxic metalloid widely distributed in environment, being responsible for mass poisoning throughout Asia^1^,^2^. In the natural environment, arsenite (As^III^) and arsenate (As^V^) are the primary arsenicals^3^,^4^, and microbial redox reactions are considered as important contributors to the changes of As^III^ and As^V^ levels^5^,^6^,^7^,^8^,^9^,^10^.

Microbial As^III^ oxidation is an elaborate regulation process^11^,^12^,^13^,^14^. The As^III^ oxidase AioBA consists of two heterologous subunits, and is responsible for catalyzing bacterial As^III^ oxidation^11^,^15^. In some As^III^-oxidizing strains, the three-component system AioXSR sensed the As^III^ signal and regulated the expression of AioBA^12^,^14^,^16^. Moreover, the phosphate two-component system PhoBR could be involved in the regulation of *aioBA* expression^13^ or bind with the promoter of *aioBA* directly^10^; The ArsR repressor, which is involved with the control of the ArsRBC arsenic detoxification system^17^,^18^ and the dissimilatory As^V^ reduction^19^, is also associated with regulation of *phoB1* gene located near the *aio* locus^13^, indicating that bacterial As^III^ oxidation was co-regulated by the *aio, pho* and *ars* regulatory systems. In addition, the As^III^/H^+^ antiporter Acr3-1 which regulated by ArsR, is essential for As^III^ oxidation, suggesting *aio, pho* and *ars* gene clusters are all involved in bacterial As^III^ oxidation^20^.

Based on the Mitchellian chemiosmotic energy conversion, the electrochemical disequilibrium between reducing and oxidizing substrates results in the electron transport via the redox reaction, associated with energy generation, which is a common feature of bacteria^21^,^22^. Microbial As^III^ oxidation is considered as a detoxification mechanism or contributes to energy generation redox reactions depending on the microorganisms^9^,^23^,^24^,^25^. In some autotrophic As^III^-oxidizing strains, NO_3_^−^ or O_2_ is the final electron acceptor of the As^III^ oxidation, assisting to generate energy to support bacterial growth^23^,^26^. A photosynthetic As^III^-oxidizing bacterium was reported to grow as a photoautotroph using As^III^ as the sole photosynthetic electron donor^27^. In addition, the heterotrophic As^III^-oxidizing bacteria *Hydrogenophaga* sp. NT-14 and *Agobacterium tumefaciens* GW4 were also reported to be able to generate energy from As^III^ oxidation^25^,^28^. *A. tumefaciens* GW4 is especially effective at improving the generation of both ATP and NADH by As^III^ oxidation^25^. Using O_2_ as the final electron acceptor, CytC was reported to be the As^III^ oxidation electron transporter with the generation of ATP^28^,^29^. However, the electron transporter for the production of NADH still unknown.

Recently, using comparative proteomics analysis, we found an oxidoreductase (named AioE) was obviously up-regulated in the presence of As^III^, as well as the As^III^ oxidation electron transporter CytC and the large subunit of As^III^ oxidase AioA. According to BlastP analysis, AioE belongs to a TrkA superfamily and contains a NAD^+^ binding domain, which could incorporate one hydroxyl group to carbonyl group by concomitant generation of NADH and H^+^ 30,31. Such function shares similarities with the reaction converting reduced AioBA back to oxidized AioBA^29^,^31^,^32^. In addition, *aioE* is located in the arsenic island containing functional *aio, pho, pst* and *ars* genes among several available arsenic islands^33^. Thus, we speculated that AioE may be important for As^III^ resistance and oxidation. Herein, the amount of ATP/NADH, the As^III^ resistance levels and As^III^ oxidation rate were compared between the *aioE* and *cytC* mutants. In addition, the redox potential of the AioAB, AioE, and CytC proteins were determined. The summarized results represent a novel contribution and demonstrate that the *aioE* is involved in As^III^ oxidation and resistance. Considering its gene function and encoding protein domains, we propose that AioE may be involved in As^III^ oxidation electron transport associating the generation of NADH.

## Results

### AioE is widely distributed in As^III^ oxidizing bacteria

**Figure 1:**
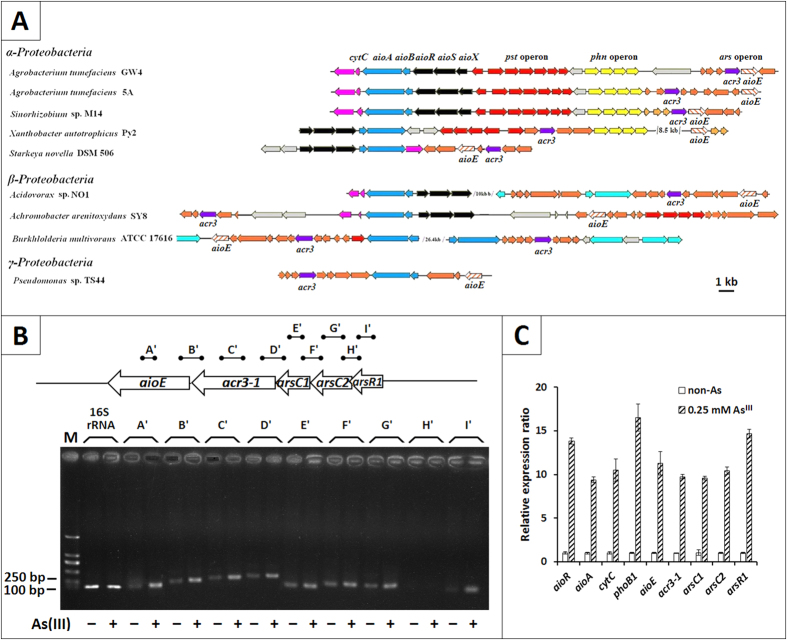
(**A**) The coding gene of oxidoreducase AioE was spread in many genomes of As^III^ oxidation strains. **(B)** RT-PCR analysis illustrating the co-transcription of *arsR1*-*arsC1-arsC2-acr3-1-aioE* and their enhanced expression with the addition of As^III^. RT-PCRs were performed with the primers in Table S1. A’, 129 bp, B’, 246 bp, C’, 274 bp, D’, 321 bp, E’, 139 bp, F’, 210 bp, G’, 226 bp, H’, 188 bp, and I’, 117 bp. Total RNA was extracted from strain GW4 grown in MMNH_4_ medium with or without 0.25 mM As^III^. **M**, the molecular weight marker (DL 2000 plus). All reverse transcriptase reactions contained 15 ng of RNA and each lane was loaded with 5 μL of the PCR product. Amplicon identities were confirmed by DNA sequencing. **(C)** Quantitative reverse transcriptase-PCR analysis of the genes involved in *ars* and *aio* gene island influenced by As^III^. The 16 S rRNA gene was used as a reference. Data are shown as the mean of three replicates, with the error bars representing ± 1 SD. Amplicon identities were confirmed by DNA sequencing.

Using comparative proteomics analysis, we found an oxioreductase, AioE, was obviously up-regulated with a 10.5 folds change in the presence of As^III^ (unpublished data) in *A. tumefaciens* GW4 (AWGV01000000). Using BlastP analysis, the AioE showed 91% amino acid identity with an oxidoreductase in *Ochrobactrum tritici* (AKB90512.1). The *aioE* gene is located at the downstream of *acr3-1* gene within the arsenic island also containing the *ars*-*pho*-*pst*-*aio* gene cluster in strain GW4 (Fig. 1A). In addition, *aioE* is widely distributed and consistently located in the arsenic islands in some As^III^-oxidizing strains (Fig. 1A), indicating that the *aioE* gene is most likely related to As^III^ resistance and oxidation. Moreover, *aioE* of the As^III^ oxidizers are phylogenetically clustered into α, β, γ-Proteobacteria (Fig. S1), which is in agreement with 16 S rRNA based phylogenetic analysis (data not shown).

### AioE is induced by As^III^ and co-transcribed with arsR1-arsC1-arsC2-acr3-1

To identify the contribution of the *aioE* gene to As^III^ resistance and oxidation, RT-PCR and qRT-PCR were employed to test the transcription level. The PCR which used DNA or RNA as the template respectively confirmed that the reagents and primers both worked well, and the results showed free of DNA contamination in the RNA (Fig. S2). The RT-PCR showed the co-transcription of *arsR1*-*arsC1-arsC2-acr3-1-aioE* (Fig. 1B), indicating that the repressor ArsR1 may regulate the transcription of *arsC1-arsC2-acr3-1-aioE*^34^,^35^. With the presence of As^III^, the transcription of *aioE* was increased by more than 10 folds, which is consistent with the proteomics data. Other genes within the *arsC2*-*arsC1*-*acr3-1*-*arsR1* operon, and As^III^ oxidation genes *aioR* and *aioA*, were also highly induced by As^III^ (Fig. 1C). In addition, the *phoB1* was also induced by As^III^, which is consistent with results in As^III^-oxidizing strain *A. tumefaciens* 5A^13^.

### AioE is essential for bacterial As^III^ resistance

**Figure 2:**
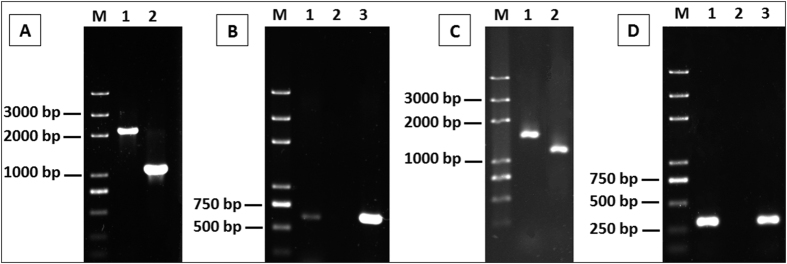
(**A**,**B**) Diagnostic PCR confirming the deletion of *aioE* to create mutant strain GW4-Δ*aioE* and complementation to create GW4-Δ*aioE*-C. (**A**) PCR amplicons using primers PaioE-1F and PaioE-2R. **(B)** PCR amplicons using primers IaioE-F and IaioE-R. **(C**,**D)** Diagnostic PCR confirming the deletion of *cytC* to create mutant strain GW4-Δ*cytC* and complementation to create GW4-Δ*cytC*-C. (**C**) PCR amplicons using primers PcytC-1F and PcytC-2R. **(D)** PCR amplicons using primers IcytC-F and IcytC-R. For panels (A and B): Lane 1, strain GW4, lane 2, *aioE* gene knock-out strain GW4-Δ*aioE* and lane 3, the complemented strain GW4-Δ*aioE*-C. For panels (C and D): Lane 1, strain GW4, lane 2, *cytC* gene knock-out strain GW4-Δ*cytC* and lane 3, the complemented strain GW4-Δ*cytC*-C. **M**, the molecular weight marker (DL 2000 plus). Amplicon identities were confirmed by DNA sequencing.

**Figure 3:**
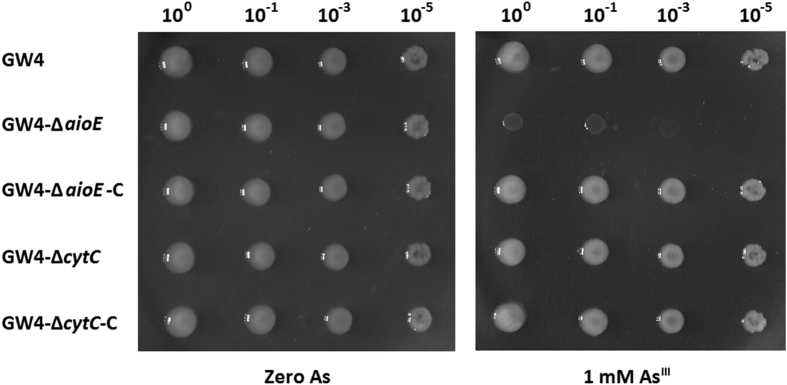
As^III^ resistance was influenced by the disruption of *aioE*. Strains GW4, GW4-Δ*aioE*, GW4-Δ*aioE*-C, GW4-Δ*cytC* and GW4-Δ*cytC*-C were inoculated in MMNH_4_ medium containing 0.1 mM phosphate. After 24 h cultivation, 10 μL of each cultures (OD_600_ = 0.5) were inoculated on the MMNH_4_ medium plate with or without the addition of 1 mM As^III^, and cultivated at 28 °C for 48 h.

To identify the function of *aioE*, we constructed *aioE* deletion mutant GW4-Δ*aioE* and its complementary strain GW4-Δ*aioE*-C (Fig. 2). In addition, in order to clarify the As^III^ oxidation electron transport function of *aioE*, we also constructed a *cytC* deletion mutant GW4-Δ*cytC* and its complementary strain GW4-Δ*cytC*-C (Fig. 2). Diagnostic PCRs (Fig. 2) and sequencing (data not shown) confirmed the successful deletion and complementation. Consistent with the decreased As^III^ resistance in *aioA* mutant^25^, the disruption of *aioE* also reduced the As^III^ resistant level (Fig. 3). However, the As^III^ resistance of mutant strain GW4-Δ*cytC* and the complementary strains were all similar to the wild type strain (Fig. 3). The results indicate that *aioE* is involved in the bacterial As^III^ resistance in strain GW4.

### AioE is essential for bacterial As^III^ oxidation

**Figure 4:**
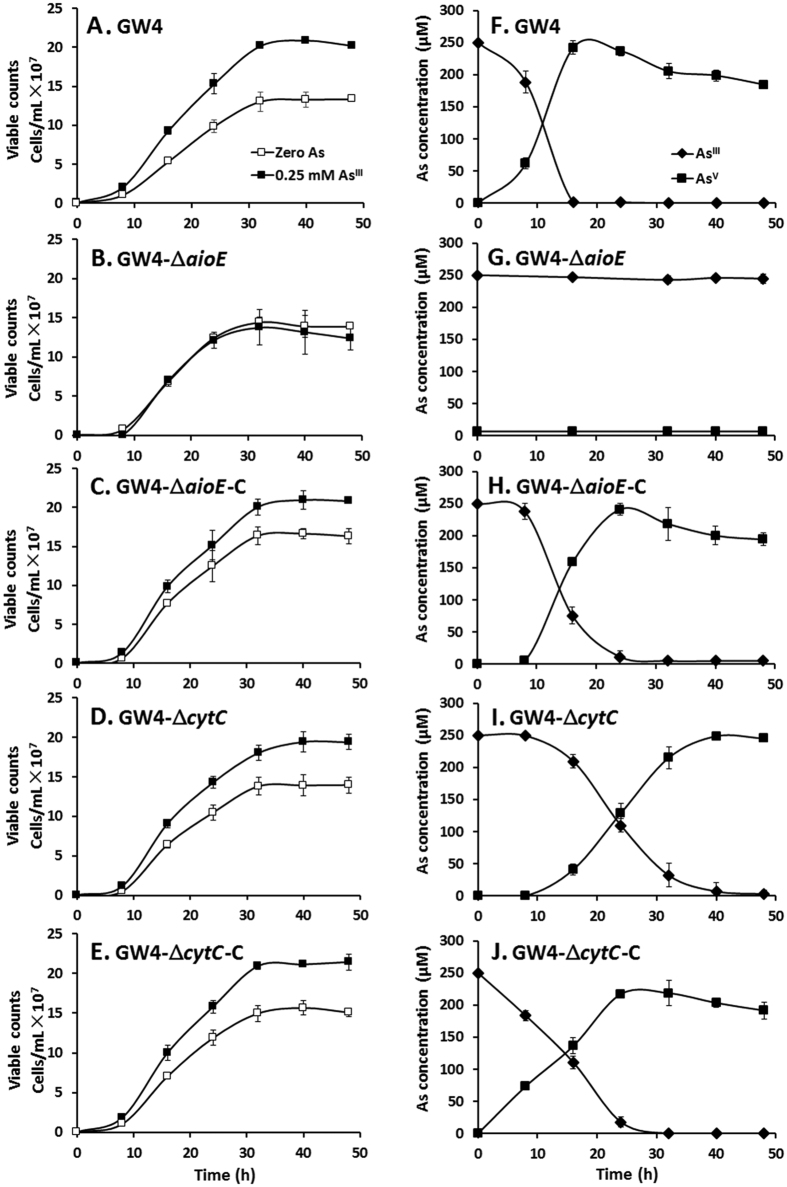
As^III^ oxidation was influenced by the disruption of *aioE*. **(A**–**E)** The growth curves of strains GW4, GW4-Δ*aioE*, GW4-Δ*aioE*-C, GW4-Δ*cytC* and GW4-Δ*cytC*-C in MMNH_4_ medium containing 0.1 mM phosphate with or without 1 mM As^III^. **(F–J)** As^III^ oxidation profiles of the same strains of B-F. As^III^ and As^V^ concentrations in the culture fluids were measured using HPLC-HG-AFS. The symbols show in panel A are the same as in panels B-E, and the symbols show in panel F are the same as in panels G-J. The data were from triplicates.

The As^III^ oxidation efficiencies of the above gene deletion and complemented strains were tested using 0.25 mM As^III^ to avoid the effect of the reduced As^III^ resistance in GW4-Δ*aioE*. The addition of 0.25 mM As^III^ resulted in enhanced growth for wild type strain GW4 (Fig. 4A), which is consistent with the previous study^25^. However, the disruption of *aioE* failed to enhance the bacterial growth with the addition of As^III^ (Fig. 4B), while the mutant strain GW4-Δ*cytC* and the complementary strains GW4-Δ*aioE*-C and GW4-Δ*cytC*-C all showed the same growth phenotype with the wild type strain GW4 in the presence of As^III^ (Fig. 4C–E). Meanwhile, consistent with the null As^III^ oxidation phenotype of deletion mutant GW4-Δ*aioA*^25^ (Fig. S3A), the disruption of *aioE* also caused in deficiency of As^III^ oxidation (Figs 4F and S3A), indicating that As^III^ oxidation was related to the enhanced bacterial growth^25^ (Fig. 4A,B,F and G). However, the mutant strain GW4-Δ*cytC* only showed a reduced As^III^ oxidation rate, and did not interrupt the bacterial As^III^ oxidation (Fig. 4I). The complementary strains of the two mutants both gained the As^III^ oxidation level back to the wild type strain (Figs 4F,H,J and S3A). The results indicated that *aioE* is essential to As^III^ oxidation and enhanced bacterial growth, and that *cytC* also participates in As^III^ oxidation, but its role is less significant compared to *aioE* in strain GW4. When *A. tumefaciens* strains grew with As^V^, GW4-Δ*aioA* and GW4-Δ*aioE* showed As^V^ reduction phenotypes, while the other *A. tumefaciens* strains failed to reduce As^V^ to As^III^ (Fig. S3B), indicating that As^V^ reduction could only occur when As^III^ oxidation is disrupted in *A. tumefaciens* GW4.

### AioE is related to the production of NADH

**Figure 5:**
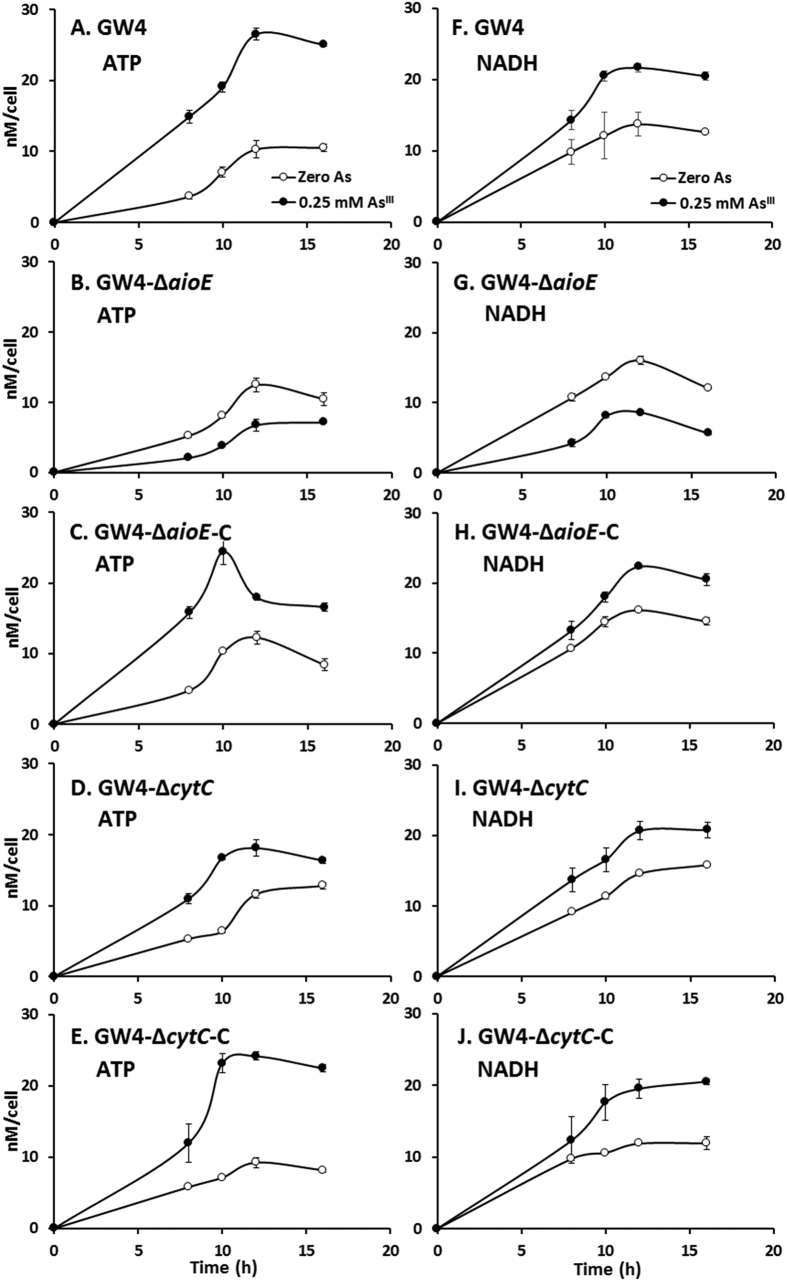
The generation of ATP and NADH was influenced by the disruption of *aioE*. **(A–E)** The ATP contents of strains GW4, GW4-Δ*aioE*, GW4-Δ*aioE*-C, GW4-Δ*cytC* and GW4-Δ*cytC*-C in MMNH_4_ medium containing 0.1 mM phosphate with or without the addition of 0.25 mM As^III^. **(F–J)** The NADH contents of strains GW4, GW4-Δ*aioE*, GW4-Δ*aioE*-C, GW4-Δ*cytC* and GW4-Δ*cytC*-C in MMNH_4_ medium containing 0.1 mM phosphate with or without 0.25 mM As^III^. The cellular contents of ATP and NADH were tested by HPLC. The symbols shown in panel A are the same as in panels B–E, and the symbols shown in panel F are the same as in panels G-I. The data were from triplicates.

In bacterial cells, NADH and ATP are produced during the electron transport process^21^,^22^. We predicted that AioE may be related to As^III^ oxidation electron transport and the generation of NADH due to the protein functional domain of AioE^29^,^30^,^31^,^32^. Thus, the NADH and ATP concentrations in the above mutant and complemented strains and the wild type GW4 were analyzed. The concentrations of cellular ATP and NADH were about 20 nM/cell with the addition of As^III^ in cells of strains GW4, GW4-Δ*aioE*-C and GW4-Δ*cytC*-C (Fig. 5); in the mutant strain GW4-Δ*aioE*, the concentrations of ATP and NADH were both decreased with the addition of As^III^ (Fig. 5B and G). However, only the concentration of ATP was reduced by the disruption of *cytC* gene (Fig. 5D), and the NADH concentration in GW4-Δ*cytC* was similar to that of strain GW4 (Fig. 5I). The tests of bacterial growth, As^III^ oxidation, and the contents of NADH/ATP revealed that *aioE* gene is involved with As^III^ oxidation and NADH generation, while *cytC* gene is related with ATP generation and had a weaker effect on As^III^ oxidation than *aioE.*

### As^III^ oxidase AioBA may transport electron to AioE during As^III^ oxidation

**Figure 6:**
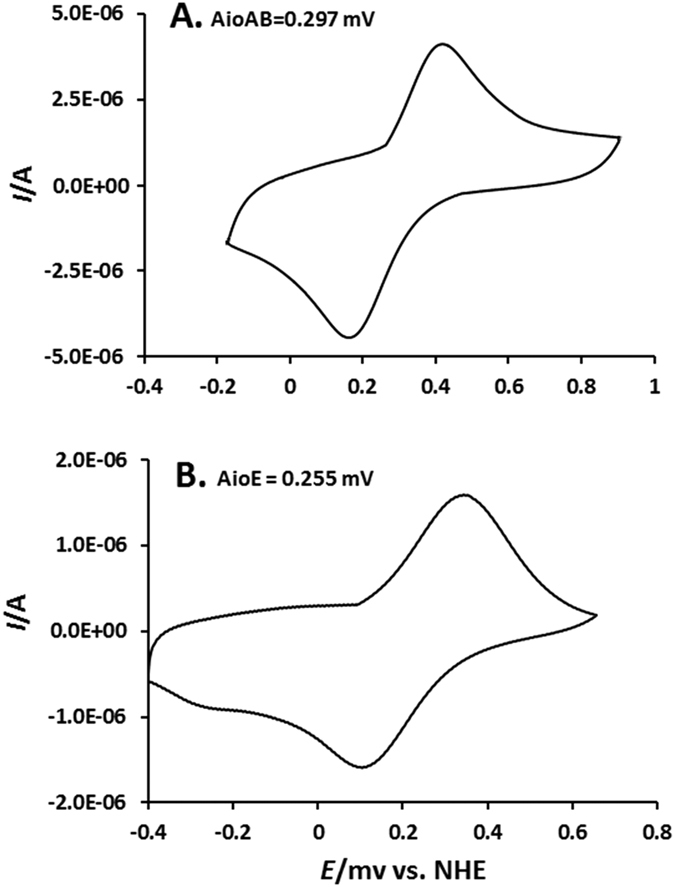
Cyclic voltammetry obtained for 4 μL of AioBA (31.5 μM, **A)** and AioE (37 μM, **B)** on Au/MUA electrode in 100 mM phosphate buffer (pH = 6) at a scan rate of 5 mV s^−1^.

To further confirm the electron transport possibility among AioBA, AioE, and CytC, the AioBA and AioE proteins were purified (Fig. S4) and their redox potentials (ORP) were obtained by cyclic voltammetry^29^. The average of the peak potentials showed the formal potential was at pH 6.0. The ORP was +0.297 V vs. NHE for AioBA, and +0.255 V vs. NHE for AioE (Fig. 6). Compared to the reported ORP of +0.217 ~ 0.251 V vs. NHE for CytC^29^,^32^, the ORP orders are AioBA > AioE > CytC. Thus, there is a possibility for AioE to participate in the electron transport between AioBA and CytC.

## Discussion

The literatures^23^,^26^,^28^ and our previous work^25^ indicate that bacterial As^III^ oxidation is not only a detoxification mechanism, but also related to the energy production. It is likely that electron transport is the cohesive tie between As^III^ oxidation and energy production^21^,^22^. In *Rhizobium* sp. NT26, *c*-type cytochrome CytC was reported as an As^III^ oxidation electron transporter^29^,^32^. Though electron transport between As^III^ oxidase AioAB and CytC was shown, the disruption of *cytC* did not cause a null phenotype for As^III^ oxidation^32^. In this study, using *A. tumefaciens* GW4, the disruption of *cytC* did not interrupt the bacterial As^III^ oxidation, but only reduced the As^III^ oxidation rate which is consistent with the results from strain NT26^32^, indicating that the As^III^ oxidation electron transport was not be completely blocked without the CytC. Thus, CytC has an additive effect on As^III^ oxidation, but is not essential^32^. This suggests that there should be another protein that can serve as the electron acceptor to the As^III^ oxidase AioBA. Herein, we found considerable evidence that *aioE* is related to the As^III^ resistance and oxidation in strain GW4. This conclusion is supported by the As^III^ induced expression of *aioE* (Fig. 1), a decrease of As^III^ resistance (Fig. 3), and interruption of As^III^ oxidation in *aioE* mutant (Fig. 4). It also indicates that *aioE* may be more essential to As^III^ oxidation than *cytC*.

Bacteria often gain energy from the electron transport between the reducing and oxidizing substrates^21^,^22^,^31^. Consistent with our previous study^25^, As^III^ improved the production of both NADH and ATP in strain GW4 (Fig. 5A and F). Being the electronic anchorman of respiratory chain, CytC has multiple copies in bacterial genomes and is reported to be able to produce ATP by transferring the electron to oxygen, as well as in As^III^ oxidation process^29^,^32^, which is correlated to the reduced cellular concentration of ATP in the *cytC* mutant (Fig. 5). Compared to the decreased cellular concentration of ATP in *cytC* mutant, the obvious decreased cellular concentrations of NADH and ATP in *aioE* mutant, and the reverted NADH and ATP concentrations in strains GW4-Δ*cytC*-C and GW4-Δ*aioE*-C (Fig. 5) indicated that AioE may be related with the generation of NADH, and CytC may be involved with the generation of ATP with the addition of As^III^. Because the ORP gradient is AioBA > AioE > CytC (Fig. 6), we infer that the electron may be transferred from AioBA to AioE with the generation of NADH, and then to CytC with the generation of ATP. This hypothesis is agreed with the BlastP predicted protein function of AioE that it produced NADH when catalyzing the reaction of hydroxyl to generate carbonyl, which is similar to the reaction converting reduced AioBA back to oxidized AioBA^29^,^31^,^32^. The AioE may be responsible to the electron transport from the molybdenum ion center of AioBA with the generation of NADH and H^+^ 29,30,31,32. Though Cytochrome C is an electronic anchorman of Complex III in the respiratory chain, so far, four Complexes of the respiratory chain have been found to be able to create an electrochemical proton gradient that drives the synthesis of ATP. Moreover, Complex I could transfer electrons from the generated NADH to produce proton gradient^36^, which could link the As^III^ oxidation with the respiratory chain, and then produce energy to support the bacterial growth (Fig. 4D).

Acting as an As^III^ induced gene (Fig. 1C), *aioE* is located in the *ars* operon including genes responsible for As^III^ oxidation and resistance in strain GW4, and co-transcribed with the *arsR1*-*arsC1-arsC2-acr3-1*. ArsR1 encoded by the *arsR*1 positioned close nearby *acr3-1* may take charge of the regulation of *aioE* expression. Being described in the literatures published so far, ArsR repressor protein was responsible for the regulation of *ars* operons invariably^37^,^38^,^39^. Meanwhile, it was also reported to function as a regulator to involve in the expression of *pstS1* and *phoB1*, which are located immediately adjacent to the *aio* gene cluster and essential for As^III^ oxidation^13^. The regulation of *aioE* expression provides more evidence for the involvement of ArsR in bacterial As^III^ oxidation.

Based on the RT-PCR results, the putative As^V^ reductase gene *arsC* and *aioE* are in the same operon. It is truly interesting that strain GW4 has both As^III^ oxidation and As^V^ reduction function genes in the same operon. When *aioE* or *aioA* was deleted, the As^III^ oxidation phenotype was disrupted and the As^V^ reduction phenotype was shown in the mutants which is most probably due to the exist of the *arsC* (Fig. S3). Generally, As^III^ oxidation is also coupled with the enhanced bacterial growth (Fig. 4) via the production of NADH and ATP (Fig. 5) in strain GW4, which is probably more effective than As^V^ reduction and efflux for bacterial arsenic resistance. This may be a reason for the As^III^ oxidation phenotype is dominant in strain GW4. In future studies, it is interesting to confirm if the ArsC is the As^V^ reductase or if this operon is regulated by ArsR. In addition, it is truly interesting to know that the two opposite function genes in the same operon is associated to the highly arsenite resistance (8 mM) of strain GW4.

In addition, being an As^III^/H^+^ antiporter, Acr3-1 is generally considered as an As^III^ resistance protein^40^, while its coding gene located in the *ars* gene clusters isregulated by ArsR^37^,^38^,^39^. Interestingly, the essential of Acr3-1 for bacterial As^III^ oxidation was discovered recently^20^, indicating that the As^III^/H^+^ antiporter on bacterial membrane was important for As^III^ oxidation. AioE may produce H^+^ when transport electron from hydroxyl^31^, thus, when AioE transport the electron from AioBA, the generated H^+^ may involve with the As^III^ trafficking across the cytoplasmic membrane, which was proven to be important to As^III^ resistance and As^III^ oxidation occurred in periplasm^20^,^23^,^28^.

In conclusion, we showed that the oxidoreductase AioE is essential for As^III^ oxidation and resistance in heterotrophic As^III^ oxidizing bacterium *A. tumefaciens* GW4. AioE appears to act as a novel electron transporter associating with the generation of NADH during bacterial As^III^ oxidation. Since As^III^ oxidation contributes the detoxification and the production of energy, the essential of AioE for As^III^ resistance is also reasonable.

## Methods and Materials

### Bacterial strains and culture conditions

Bacterial strains and plasmids used in this study are listed in Table S1. *A. tumefaciens* strains were grown in a defined minimal mannitol medium (MMNH_4_)^41^ at 28 °C containing 0.1 mM phosphate, with or without the presence of 0.25 mM NaAsO_2_ (As^III^). *E. coli* strains were grown in Luria-Bertani medium^42^ at 37 °C. When necessary, kanamycin (Kan, 50 μg/mL), gentamicin (Gen, 50 μg/mL), tetracycline (Tet, 5 μg/mL) or ampicillin (Amp, 100 μg/mL) was added.

### Phylogenetic relationship analysis

The *aioE* sequences was downloaded from National Center for Biotechnology Information Search database (NCBI). Phylogenetic relationships based on neighbor-joining method were then examined by downloading and aligning various sequences using ClustalX v1.83^43^ with tree constructed using Mega 6.0^44^.

### RT-PCR and quantitative RT-PCR analysis

Overnight cultures of strain GW4 were inoculated into 100 mL MMNH_4_ medium with or without the addition of 0.25 mM As^III^ respectively and incubated at 28 °C with 100 rpm shaking. Samples used for RNA isolation were taken after 16 h cultivation. Total RNA was extracted used Trizol Kit (Invitrogen) and incubated with RNase-free DNase I (Takara) at 37 °C to remove the genomic DNA. Then, the reaction was terminated by addition of 50 mM EDTA at 65 °C for 10 min^45^. After confirming the negative DNA contamination and determining the concentration of RNA by spectrophotometer (NanoDrop 2000, Thermo), RT-PCR for testing the co-transcribe of the *ars* gene cluster was performed using the primers listed in Table S2, while 300 ng total RNA was reverse transcribed into cDNA with RevertAid First Strand cDNA Synthesis Kit (Thermo). The obtained cDNA was diluted 10-folds for real-time RT-PCR analysis using SYBR® Green Realtime PCR Master Mix (Toyobo)^46^ with primers listed in Table S2. Quantitative RT-PCR was performed by ABI VIIA7 in 0.1 mL Fast Optical 96-well Reaction Plate (ABI). Each reaction was replicated three times for eliminating the error. Gene expression was normalized by ∆∆CT analysis with an iQ5 Real-Time PCR Detection System (Bio-Rad, USA). All of the PCR products were confirmed by sequencing.

### Construction of aioE and cytC mutant and complementation strains

The in-frame deletion in *aioE* and *cytC* was respectively constructed using crossover PCR^47^ with primers listed in Table S2. The PCR products were both cloned into *BamH*I and *Xba*I double digested pJQ200SK, respectively. The final constructed pJQ-*aioE* and pJQ-*cytC* were separately mobilized into GW4 via conjugation with *E. coli* strain S17-1. Single cross-over mutants of *aioE* or *cytC* were identified on MMNH_4_ agar plate containing 50 μg/mL Gen, which were then screened on MMNH_4_ agar with 20% sucrose25^48^. Sucrose^R^ Gen^Sen^ trans-conjugants were then screened using diagnostic PCR and DNA sequencing to identify a double recombinant GW4-Δ*aioE* and GW4-Δ*cytC*.

For complementation, the complete *aioE* or *cytC* coding region was PCR-cloned as *BamH*I-*Xba*I fragments into palsmid pCPP30, respectively. Using conjugation, the resulting plasmids pCPP30-*aioE* was transferred into the mutants GW4-Δ*aioE*, while and pCPP30-*cytC* was transferred into the mutants GW4-Δ*cytC*. The mutant and complementary strains were confirmed by PCR using primers listed in Table S2 along with diagnostic sequencing. The successful complementary strain GW4-Δ*aioE*-C was constructed^49^.

### Analysis of As^III^ resistance and oxidation

To investigate the As^III^ resistance of mutant strains, overnight cultures of GW4, GW4-Δ*aioE*, GW4-Δ*aioE*-C, GW4-Δ*cytC*, and GW4-Δ*cytC*-C (OD_600_ = 0.5–0.6) in MMNH_4_ medium and three diluted concentrations of these strains were each plated (2 μL) on solid MMNH_4_ medium containing 0 or 1 mM As^III^. Plates were photographed after 2–3 days at 28 °C until colonies formed. The qualitative As^III^ oxidation was performed using AgNO_3_ staining^14^. Overnight cultures of GW4, GW4-Δ*aioE*, GW4-Δ*aioE*-C, GW4-Δ*cytC*, GW4-Δ*cytC*-C the As^III^ oxidase large subunit gene *aioA* mutant and its complementary strain GW4-Δ*aioA* and GW4-Δ*aioA*-C constructed in previous work^25^ were inoculated on MMNH_4_ agar plates containing 0.1 mM phosphate and 0.25 mM As^III^. After 48 h cultured at 28 °C, the plates were flooded with 0.1 M AgNO_3_^14^. As^V^ compounds react with AgNO3 generates brown color colonies indicating As^III^ oxidation positive, while As^III^ compounds cannot react with AgNO3 to generate brown color product revealing As^III^ oxidation negative. The quantitative As^III^ oxidation tests were detected using HPLC-HG-AFS (Beijing Titan Instruments Co., Ltd.)^25^. Overnight cultures of GW4, GW4-Δ*aioE*, GW4-Δ*aioE*-C, GW4-Δ*cytC* and GW4-Δ*cytC*-C (OD_600_ = 0.5–0.6) were each inoculated (200 μL) into 100 mL MMNH_4_ with or without 0.25 mM As^III^ and incubated at 28 °C for 48 h with 100 rpm shaking. At designated times, culture samples were taken for viable plate counts and for monitoring As^III^/As^V^. The qualitative As^V^ oxidation was performed using KMnO_4_ staining^36^. Overnight cultures of *A. tumefaciens* strains were inoculated on MMNH_4_ liquid medium containing 0.1 mM phosphate and 1 mM As^V^. After 48 h cultured at 28 °C, 1 mL cultures was mixed with 50 μL 10 mM KMnO_4_ to detect the presence of As^III^ associated with As^V^ reduction (yellow) or the absence of As^V^ reduction (pink)^36^.

### Analysis of the amount of ATP and NADH

*A. tumefaciens* GW4, GW4-Δ*aioE*, GW4-Δ*cytC* and the complementary strains were each inoculated into 100 mL MMNH_4_ medium with or without the addition of 0.25 mM As^III^ and incubated at 28 °C with 100 rpm shaking. The bacterial cells were collected by centrifugation (12,600 × g, 5 min, 4 °C) at designated times (during the As^III^ oxidation process) and resuspended in 1 mL 0.4 M perchloric acid with 1.0 mM EDTA. After 5 min ultra-sonicated on ice, the unbroken cells were removed by centrifugation (12,600 × g, 5 min, 4 °C). Then the pH of the extracts were adjusted to 7.0 with 1 M K_2_CO_3_ and percolated with 0.22 μm filter membrane. The samples were analyzed by HPLC (HPLC 2690 series, Waters, Massachusetts, USA), using the mobile phase containing 90% 50 mM phosphate buffer, 10% acetonitrile, and 3.22 g/L tetrabutylammonium bromide (pH 6.8), and the flow velocity of the mobile phase was 1 mL/min. The amount of ATP and NADH were measured by comparing the retention times to standards^50^.

### Expression and purification of proteins

The AioAB and AioE proteins were expressed using *E. coli* BL21 Star^TM^ (DE3) pLysS for *aioAB* on vector pPROEX-HTA and *aioE* on vector pET-32a(+), respectively. Cells were grown at 37 °C overnight in LB medium containing the required antibiotics. Overnight culture was inoculated into 100 mL of LB and the culture was grown to OD_600_ of 0.1 and induced with 0.02 mM isopropyl-β-d-thiogalactoside (IPTG) for 16 h. Cells were collected by centrifugation (8,000 r/min for 10 min at 4 °C) after induction, and resuspended in 50 mM Tris-HCl (pH 7.5). After lysed by high pressure cell cracker and centrifuged at 8,000 rpm for 10 min at 4 °C, the cleared lysate of AioBA or AioE was respectively applied on a column of pre-equilibrate ProfinityTM IMAC Resins (Bio-RAD) by gravity flow. Each column was washed with 3 mL of Tris-HCl containing 20 mM imidazole (pH 7.5). Then AioE was eluted with Tris-HCl containing 200 mM imidazole (pH = 7.5), while AioAB was eluted with Tris-HCl containing 40 mM imidazole (pH 7.5). Purified proteins AioAB and AioE were stored at −80 °C, when used, the eluate was dialyzed against PBS to remove imidazole^14^. The concentrations of the purified AioBA and AioE were determined by Nano Drop 2000 (Thermo Scientific).

### Detection of the redox potencial (ORP) of proteins using cyclic voltammetry

The ORP of the proteins were tested using cyclic voltammetry (CV) experiments, performed in PBS buffer, pH = 7.0, at 16 °C using a BAS 100B/W electrochemical workstation coupled with a BAS RDE-3 rotating disk electrode cell stand^32^. A three-electrode system was employed comprising a gold working electrode, a Pt wire counter, incorporating a saturated calomel electrode (SCE) as the reference. The experiments were carried out with 60 min nitrogen purged solutions and a nitrogen blanket was maintained during the measurement. The Au working electrode was mechanically, chemically, and electrochemically cleaned and polished as described^51^. The monolayer of 11-mercaptoundecanoic acid (MUA) was prepared on a clean Au electrode by immersion in a 20 mM ethanolic solution of MUA for at least 24 h^52^. The electrode was subsequently washed with copious amounts of ethanol and water to remove any loosely bound MUA molecules from the electrode surface. The electrode was placed in a solution containing 4 μL AioBA (31.5 μM) or AioE (37 μM) for 16 h at 4 °C, then it was rinsed with PBS buffer (pH = 7.0) to remove all protein molecules that were not immobilized on the surface. The experimental cyclic voltammograms (CVs) were simulated with the Chi660 program^53^.

## Additional Information

**How to cite this article**: Wang, Q. *et al*. An Oxidoreductase AioE is Responsible for Bacterial Arsenite Oxidation and Resistance. *Sci. Rep.* **7**, 41536; doi: 10.1038/srep41536 (2017).

**Publisher's note:** Springer Nature remains neutral with regard to jurisdictional claims in published maps and institutional affiliations.

## Supplementary Material

Supplementary Information
