# Supplementary material for: An Oxidoreductase AioE is Responsible for Bacterial Arsenite Oxidation and Resistance
Source: Sci Rep. 2017 Jan 27;7:41536. doi: 10.1038/srep41536 (PMC5270249; doi:10.1038/srep41536)
Supplement: Supplementary Information [file srep41536-s1.pdf]

An Oxidoreductase AioE is Responsible for Bacterial Arsenite Oxidation and Resistance

Qian Wang, Yushan Han, Kaixiang Shi, Xia Fan, Lu Wang, Mingshun Li,

and Gejiao Wang*

State Key Laboratory of Agricultural Microbiology, College of Life Science and Technology, Huazhong Agricultural University, Wuhan 430070, P. R. China

Corresponding Authors:

Gejiao Wang, State Key Laboratory of Agricultural Microbiology, Huazhong Agricultural University, Wuhan 430070, China, Phone: 86-27-87281261; E-mail: gejiao@mail.hzau.edu.cn

Running title: Oxidoreductase involved in As^{III} oxidation

Figure S1

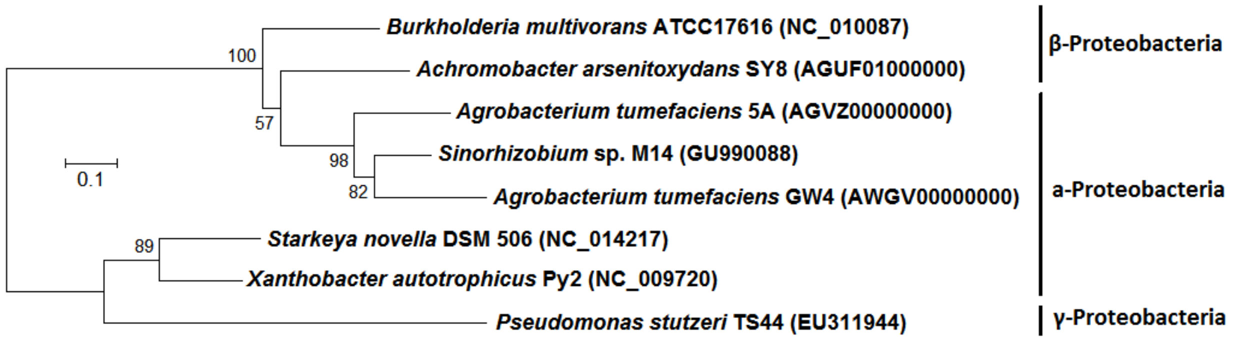

Figure S1. Un-rooted neighbor-joining tree illustrating the phylogenetic relationships of *aioE* gene in different As^{III} oxidizers showed in Fig. 1A. The bootstrap values were generated from 500 pseudoreplicates.

Figure S2

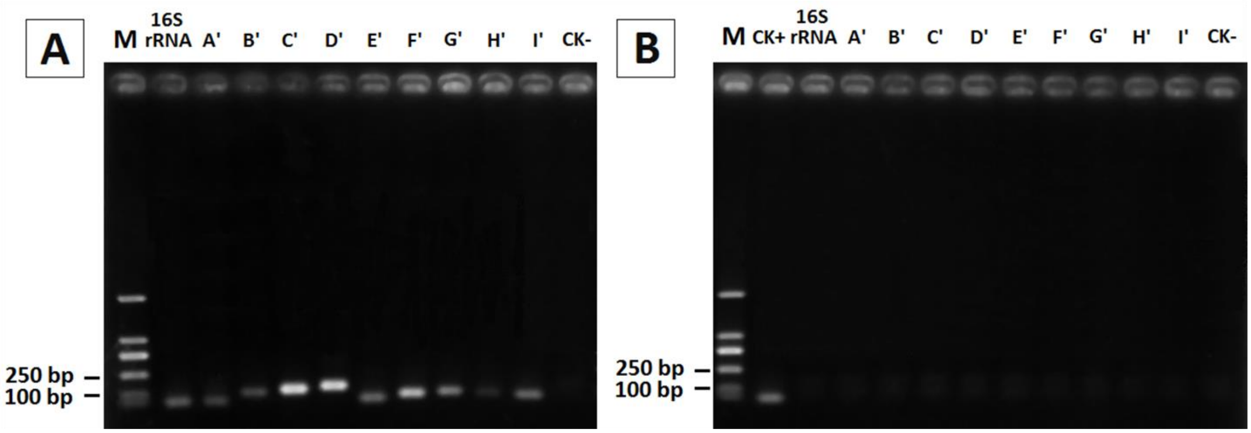

Figure S2. The products of PCR using DNA (**A**) or RNA (**B**) as template using the primers showed in Fig. 1B. The primers were performed with the primers in Table S2. A', 129 bp, B', 246 bp, C', 274 bp, D', 321 bp, E', 139 bp, F', 210 bp, G', 226 bp, H', 188 bp, and I', 117 bp. Total RNA was extracted from strain GW4 grown in $MMNH_4$ medium with or without 0.25 mM As(III). **M**, the molecular weight marker (DL 2000 plus). Amplicon identities were confirmed by DNA sequencing.

Figure S3

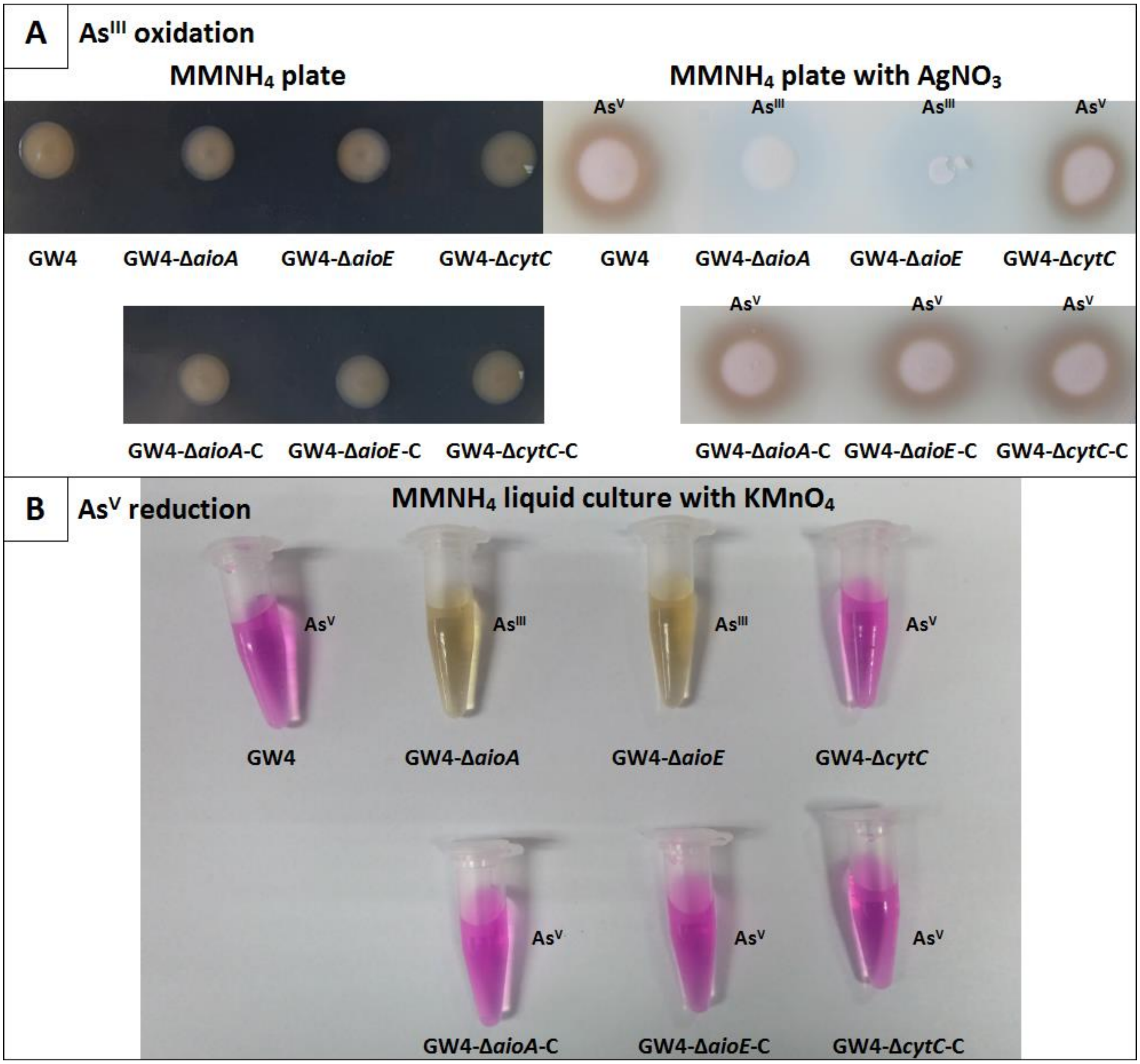

Figure S3. (A) Qualitative analysis of As^{III} oxidation using $AgNO_3$ staining. *A. tumefaciens* strains GW4, GW4-Δ*aioA*, GW4-Δ*aioA*-C, GW4-Δ*aioE*, GW4-Δ*aioE*-C, GW4-Δ*cytC*, and GW4-Δ*cytC*-C were inoculated onto the $MMNH_4$ agar plates containing 100 µM Pi and 0.25 mM As^{III}. After 48 h incubation, 0.1 M $AgNO_3$ was flooded onto the plates. Brown precipitate indicates the presence of As^{V}, whereas yellow indicates As^{III}. **(B)** Qualitative analysis of As^{V} reduction using $KMnO_4$ staining. *A. tumefaciens* strains GW4, GW4-Δ*aioA*, GW4-Δ*aioA*-C, GW4-Δ*aioE*, GW4-Δ*aioE*-C, GW4-Δ*cytC*, and GW4-Δ*cytC*-C were inoculated onto the $MMNH_4$ liquid medium containing 100 µM Pi and 1 mM As^{V}. After 48 h incubation, 50 µL 0.1 mM $KMnO_4$ was added into 1 mL cultures. Pink indicates the presence of As^{V}, while yellow indicates As^{III}. The As valences of the cultures are labeled.

Figure S4

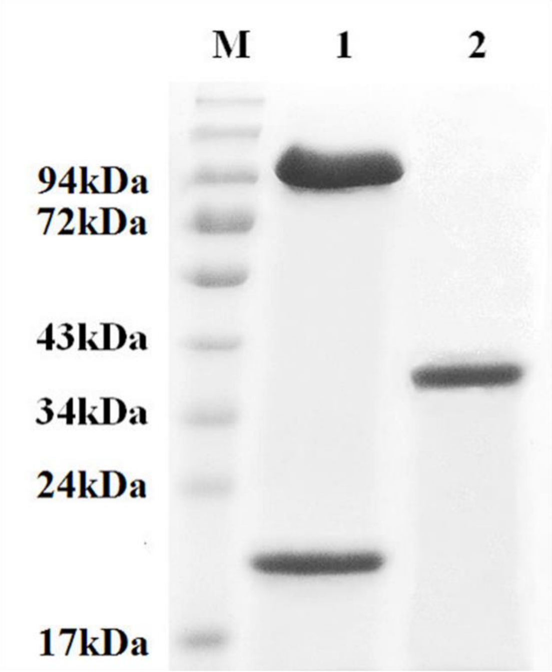

Figure S4. The purification of AioAB and AioE in *A. tumefaciens* GW4. AioBA and AioE were over expressed with a His_6 tag in *E. coli* BL21. Lane 1, purified AioAB; Lane 2, purified AioE.

Table S1. The strains and plasmids used in this research.

Strain/plasmid	Relevant properties or derivation	Source or reference
**Strains**		
Agrobacterium tumefaciens		
GW4	Wild type, As^{III} oxidizing strain	Fan *et al* ., 2008
GW4-Δ*aioA*	*aioA* gene deleted	Wang et al., 2015
GW4-Δ*aioA*- C	Complementation of GW4-Δ*aioA*	Wang et al., 2015
GW4-Δ*aioE*	*aioE* gene deleted	This research
GW4-Δ*aioE*- C	Complementation of GW4-Δ*aioE*	This research
GW4-Δ*cytC*	*cytC* gene deleted	This research
GW4-Δ*cytC* -C	Complementation of GW4-Δ*cytC*	This research
Escherichia coli		
DH5α	*supE* 44 *lacU* 169(*φ80lacZM15*) *hRDR* 17 *recA* 1 *endA* 1 *gyrA96 thi-1 relA* 1	Hanahan, 1983
BL21Star^{TM}(DE3)pLysS	F^- *ompT hsdSB* (r_B^- m_B^-) *gal dcm me* 131 (DE3) pLysS (Cm^R)	Invitrogen
S17-1 λ*pir*	F^- RP4-2-Tc::Mu *aphA* ::Tn7 *recA* λ*pir* lysogen; Sm^R Tp^R	Simon et al., 1983
**Plasmids**		
pGEM-T	TA cloning vector; Amp^R	Promega
pJQ200SK	*sacB sac* ^{R} Suc^S; Gen^R	Quandt and Hynes, 1993
pJQ-*aioE*	Gene mutation plasmid to create GW4-Δ*aioE* ; *sacB sac* ^{R} Suc^S; Gen^R	This research
pJQ-*cytC*	Gene mutation plasmid to create GW4-Δ*cytC* ; *sacB sac* ^{R} Suc^S; Gen^R	This research
pCPP30	Broad host range; Tet^R	Liu et al., 2012
pCPP30(*aioE*)	Complementing plasmid for GW4-Δ*aioE* ; Tet^R	This research
pCPP30(*cytC*)	Complementing plasmid for GW4-Δ*cytC* ; Tet^R	This research
pProEX-Hta	*trc* promotor expression vector; Amp^R	Novagen
pProEX-Hta(*aioAB*)	*aioAB* gene cloned into pProEX-Hta; Amp^R	This research
pET-32a(+)	T7 promotor expression vectpr; Amp^R	Novagen
pET-32a(+)(*aioE*)	*aioE* gene cloned into pProEX-Hta; Amp^R	This research

Table S2. Primers used in this research.

Primer pair	Primer sequence	Use
PaioE-1F/<br>PaioE-1R	5'AAATCTAGACGTTTACGCCTGGTTCTT3'/<br>5'**CTTCTCGTGATCGTATCGTCA**CTCCCCGTTTTATTCTGC3'	Crossover PCR to create deletion in *aioE*
PaioE-2F/<br>PaioE-2R	5'**TGACGATACGATCACGAGAAG**CTTAGTTGGGCTGGCTGCTG3'/<br>5'AAAGGATCCTTGGCTTGGACTTTCATTTCTG3'	Crossover PCR to create deletion in *aioE*
PcytC-1F/<br>PcytC-1R	5'AAATCTAGACCACGGATGATGGAAAGG3'/<br>5'**ATCGGCGCCATAAATGCGCAT**AACGACAATGTTGCGAGAAA3'	Crossover PCR to create deletion in *cytC*
PcytC-2F/<br>PcytC-2R	5'**ATGCGCATTTATGGCGCCGAT**GTGGGCACCGTCATCCT3'/<br>5'AAAGGATCCCTGCGACCGTTCCTTCTAC3'	Crossover PCR to create deletion in *cytC*
CaioE-F/<br>CaioE-R	5'AAATCTAGACGCCGCGTCATCTAGTCT3'/<br>5'AAAGGATCCGCACCTGCTCGCTCATTT3'	Complementation for *aioE* mutant
CcytC-F/<br>CcytC-R	5'AAACTGCAGATGGCTTTCAAACGTCACG3'/<br>5'AAAGGATCCTATTCCGTGAGGTGGCTCT3'	Complementation for *cytC* mutant
IaioE-F/<br>IaioE-R	5'TCAGAAGCGTTGATGGGACT3'/<br>5'GGTTGTTTCAGCGATTGTCC3'	Confirm for GW4-Δ*aioE* and GW4-Δ*aioE* -C
IcytC-F/<br>IcytC-R	5'AAACTGCAGATGGCTTTCAAACGTCACG3'/<br>5'AAAGGATCCTATTCCGTGAGGTGGCTCT3'	Confirm for GW4-Δ*cytC* and GW4-Δ*cytC* -C
RT16S-F/<br>RT16S-R	5'GGTATGGGCATTGGAGACGA3'/<br>5'GGCAACTAAGGGCGAGGG3'	RT-PCR and qRT-PCR for 16S rRNA gene
RTaioE-F/<br>RTaioE-R	5'TGTGGATTCGGTCATCTGGTG3'/<br>5'CAAAGGCGAGGTTGTTTCAGC3'	RT-PCR for *aioE*
RT-H3-F/<br>RT-H3-R	5'CGACGCAGGTAGTAGGC3'/<br>5'CCATCGCAGCATTCG3'	test the cotranscription of *depH* and *acr3*
RTacr3-F/<br>RTacr3-R	5'CAAGCGTGTCCTCGGTAT3'/<br>5'AGGCGGCAAGAAAGTAAG3'	RT-PCR for *acr3*
RT-3c1-F/<br>RT-3c1-R	5'CGAGGAAAGATAGGCG3'/<br>5'CGGATGAGCACCTTGT3'	test the cotranscription of *acr3* and *arsC1*
RTarsC1-F/<br>RTarsC1-R	5'GGAGCATCCTATTCTCATCAACC3'/<br>5'GACGACCTCACCATCTTCCTT3'	RT-PCR for *arsC1*
RT-C1C2-F/<br>RT-C1C2-R	5'ATTCGGGATTGTGGTAGA3'/<br>5'AGCCCTGAACGGACTT3'	test the cotranscription of *arsC1* and *arsC2*
RTarsC2-F/<br>RTarsC2-R	5'GCCAAAGGGCGAAGTCAA3'/<br>5'TGTAGCGGCTGCGGGAT3'	RT-PCR for *arsC2*
RT-C2R-F/<br>RT-C2R-R	5'TTGACTTCGCCCTTTGG3'/<br>5'CATCGCTGAACTCACTCC3'	test the cotranscription of *arsC2* and *arsR*

RTarsR-F/<br>RTarsR-R	5'TTGCCCGTGCGCTTATC3'/<br>5'TGCCGCCACAACAATCC3'	RT-PCR for *arsR*
qRTaioR-F/<br>qRTaioR-R	5'GCGATACCGCAACACCTT3'/<br>5'GCCGTTTGGCTCATTCTG3'	qRT-PCR for *aioR*
qRTaioA-F/<br>qRTaioA-R	5'ATGCCAGACTGCCTGATTG3'/<br>5'GTGCTTGTTGTAACCGTCCA3'	qRT-PCR for *aioA*
qRTcytC-F/<br>qRTcytC-R	5'CAACTACTCCGCAGCCTTCA3'/<br>5'CATCTTGGTGCCTGGGACATA3'	qRT-PCR for *cytC*
qRTphoB1-F/<br>qRTphoB1-R	5'CTCCTCCGCTATAATCTTG3'/<br>5'GGCAGTCGTTCAGTATT3'	qRT-PCR for *phoB1*
qRTaioE-F/<br>qRTaioE-R	5'TGTGGATTCGGTCATCTGGTG3'/<br>5'CAAAGGCGAGGTTGTTTCAGC3'	qRT-PCR for *aioE*
qRTacr3-1-F/<br>qRTacr3-1-R	5'GATGATGTATCCACCTTTAGCA3'/<br>5'ACAGAGGCGGCAAGAAA3'	qRT-PCR for *acr3-1*
qRTarsC1-F/<br>qRTarsC1-R	5'CAAAGGGCGAAGTCAATCC3'/<br>5'CAACGGCAGGCTGAGAAA3'	qRT-PCR for *arsC1*
qRTarsC2-F/<br>qRTarsC2-R	5'GCCATGATCCGCAACG3'/<br>5'GACGACCTCACCATCTTCCTT3'	qRT-PCR for *arsC2*
qRTarsR1-F/<br>qRTarsR1-R	5'TTGCCCGTGCGCTTATC3'/<br>5'GCCGCCACAACAATCC3'	qRT-PCR for *arsR*
EaioE-F/<br>EaioE-R	5'AAA<u>GAATTC</u>ATGACGACCATCCACCCC3'/<br>5'AAA<u>AAGCTT</u>CGGCACCCGTAAAATCTG3	Cloning of *aioE* for over-expression of AioE
EaioAB-F/<br>EaioAB-R	5'AAA<u>GAATTC</u>GTGGCCGGGGTCGAGTAT3'/<br>5'AAA<u>AAGCTT</u>TCAGAGCGAACGGTATTC3'	Cloning of *aioAB* for over-expression of AioAB

* The bolded sequences denote the reverse complement sequences for the crossover PCR, while the underlined sequence denotes the restriction enzyme sites.
